# Early stimulation of the left posterior parietal cortex promotes representation change in problem solving

**DOI:** 10.1038/s41598-019-52668-7

**Published:** 2019-11-11

**Authors:** Ursula Debarnot, Sophie Schlatter, Julien Monteil, Aymeric Guillot

**Affiliations:** 1 Inter-University Laboratory of Human Movement Biology-EA 7424, University of Lyon, University Claude Bernard Lyon 1, Villeurbanne, France; 2 Institut Universitaire de France, Paris, France

**Keywords:** Problem solving, Cortex

## Abstract

When you suddenly understand how to solve a problem through an original and efficient strategy, you experience the so-called “Eureka” effect. The appearance of insight usually occurs after setting the problem aside for a brief period of time (i.e. incubation), thereby promoting unconscious and novel associations on problem-related representations leading to a new and efficient solving strategy. The left posterior parietal cortex (lPPC) has been showed to support insight in problem solving, when this region is activated during the initial representations of the task. The PPC is further activated during the next incubation period when the mind starts to wander. The aim of this study was to investigate whether stimulating the lPPC, either during the initial training on the problem or the incubation period, might enhance representation change in problem solving. To address this question, participants performed the Number Reduction Task (NRT, convergent problem-solving), while excitatory or sham (placebo) transcranial direct current stimulation (tDCS) was applied over the lPPC. The stimulation was delivered either during the initial problem representation or during the subsequent incubation period. Impressively, almost all participants (94%) with excitatory tDCS during the initial training gained representational change in problem solving, compared to only 39% in the incubation period and 33% in the sham groups. We conclude that the lPPC plays a role during the initial problem representation, which may be considerably strengthened by means of short brain stimulation.

## Introduction

From the famous “Eureka” effect illustrated on scientific discoveries to those experienced in everyday life, insight is one of the most fascinating human assets^1^. The existing body of research has established that insight is a sudden change in the type of knowledge representation leading to a new, effective solution or solution strategy^1,2^. Classically, insight has been opposed to analytical strategy whereby individuals proceed deliberately through the problem step-by-step, while conscious of their progress toward a solution^3^. The Number Reduction Task (NRT) is a convergent problem solving enabling the assessment of both solving strategies by means of objective and subjective outputs^4,5^. Most insights likely occur from two successive states whereby an initial and conscious work on a problem solving is left aside across an incubation period, thereby promoting unconscious and novel associations on problem-related representations^6^. Accordingly, numerous NRT studies reported that either a night of sleep^7,8^, or even a short period of quiet rest^5^, potentiate representational change in problem solving, hence promoting insight strategy.

A recent neuroimaging meta-analysis highlighted that working on problem solving followed by an incubation period might be underpinned by an antagonistic, albeit cooperative, interaction between the executive control and default mode networks^9^. The former is associated to cognitive processes that require externally directed attention, including working memory and task-set switching, whereas the latter is activated without external engagement on an attentional demanding task^10,11^. Previous evidence underlined the critical role played by the posterior parietal cortex (PPC) during both executive control and default mode networks, presumably due to its roles in attentional processes, cognitive control, task rule interpretation, and maintenance^12,13^. Interestingly, specific activity in the left PPC (lPPC) at initial practice on the NRT has been identified as a neural precursor of delayed representational change in problem solving^14–16^. Specifically, Lang *et al*.^15^ suggested that a larger amplitude of slow wave activity in the lPPC during initial training on the NRT in future insight solver, compared to analytical solvers, might contribute to a deeper encoding of knowledge in short-term memory. Although not reported in insight problem solving, the PPC has been further reported to remain active when the mind wanders during creative incubation periods^17,18^. Supporting results on the role of this region in insight problem solving were observed using anodal (i.e. excitatory) transcranial direct current stimulations (a-tDCS) over both right and left PPC before practice on a convergent verbal problem solving (i.e. compound remote task)^19^. Depolarization of the PPC was supposed to alleviate bottom-up automatic mechanisms, hence fostering insight solutions. Yet and although tDCS already demonstrated beneficial effects on insight problem solving^20,21^, the optimal montage and timing of stimulation delivery remained understudied^22^.

The present study thus aimed to test how and when substantially boosting representational change in problem solving. To address this question, we tested whether unilateral a-tDCS over the lPPC during either initial practice on the NRT, or the immediate subsequent incubation period, would substantially potentiate the emergence of insight solving strategy.

## Material and Methods

### Participants

Fifty-four healthy adults (mean age 21.8 ± 1.9 years; 29 women) voluntarily participated in this study. The a priori definition of appropriate sample sizes was based on effect sizes reported in the relevant literature using tDCS for similar group comparisons^19,23^. Participants were evenly and randomly assigned to three experimental groups (*n* = 18 in each group) according to the type and the timing of brain stimulation delivery during the task, namely the TrainStim group (a-tDCS during initial training), the ShamStim group (sham-tDCS during initial training), and the IncubStim group (a-tDCS during incubation). All were right-handed, as assessed by the Edinburgh Handedness Inventory^24^. Prior history of drug or alcohol abuse, neurological, musculoskeletal, psychiatric or sleep disorders, constituted exclusion criteria. This study was approved by the Research Ethics Committee of the Center of Research and Innovation in Sport (University Claude Bernard Lyon 1). All participants signed an informed consent form in agreement with the terms of the Declaration of Helsinki (2013). The experiment was performed in accordance with relevant guidelines and regulations. Participants were not aware of the aims and hypotheses of the study.

### Experimental procedure

**Figure 1:**
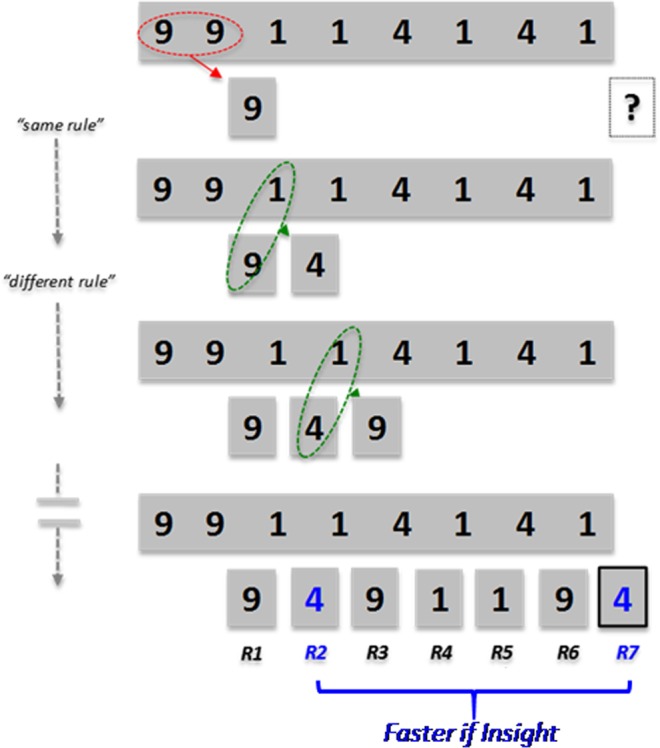
Number Reduction Task (NRT). Each stimulus string was always composed by digits ‘1’, ‘4’ and ‘9’. Participants were asked to find the final response (R7) corresponding to the solution. To do so, participants might perform sequential processing of digits pairwise from the left to the right according to two simple rules: the ‘same rule’, which states that the result of two identical digits result in the same digit (response 1 here); the ‘different rule’, which states that the result of two non-identical digits is the remaining third digit (responses 2 and 3 here). Critically, the mirror pattern present in the stimulus string (1 4 1 4 1 here) is reflected in the pattern of response string such as responses 2–4 always mirror responses 5–7, hence the second digit response is systematically the final solution. Participants who gain insight of this hidden rule through change in the NRT representation show an abrupt and significant decrease in response time to give the correct solution (R7), and in doing so press systematically the space bare at a significant earlier step (R2). By contrast, analytical participants proceed through the problem step-by-step using the same and different rules toward the solution.

We used the well-known NRT, which advantageously provides an objective and reproducible assessment of both insight and analytical solving strategies (see detailed methodology in Debarnot *et al*.^4^). This task is constituted by a series of eight numbers that must be sequentially combined following initial rules to obtain a final numeric solution (Fig. 1). Importantly, participants were not aware that all strings contain the same underlying pattern with the last three responses mirroring the three preceding ones. This pattern allows the second response to be identical to the final one, and offers (when discovered) an efficient strategy to reach the solution from the very early steps in the series. Thus, participants may either apply the initial rules step-by-step (i.e. analytic strategy), or explicitly discover the hidden rule, by change in the representation of the NRT, which was reflected by a collapse into response times to enter the correct solution (i.e. insight strategy).

**Figure 2:**
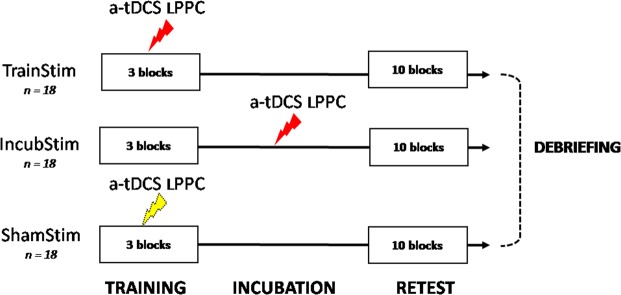
Experimental design. Concomitant a-tDCS was applied over the lPPC either during training (three blocks) or the incubation period. Excitatory stimulation at 2 mA during 19 min was applied in the TrainStim and the IncubStim groups, while false stimulation (0 mA during 19 min) was applied in the ShamStim group. No further stimulation was performed during retest (10 blocks). Finally, during the debriefing, participants were asked to explain how they proceeded during the task.

Before the experiment, all participants were equipped with the same tDCS montage, but parameters and timing of stimulation delivery were different (Fig. 2). Standardized NRT instructions were given by the experimenter, and included a short practice block of five trials to ensure the correct understanding of the initial rules and goal of the task. Then, all participants performed an initial training session on the NRT during 3 blocks, which was followed by an incubation period whereby participants were asked to remain quietly seated and relaxed during 20 min. Finally, they performed a retest session that included 10 blocks of 30 trials each. Importantly, whenever the experimenter noticed a short cut to find the correct solution number in using systematically the space bare earlier at each trial (i.e. the second response number), an additional block of practice was performed to verify the representational change from analytical to insight solving strategy. Right after the retest, two questions were systematically asked to participants: “how did you proceed during the task”, and “what did you do exactly”. This debriefing was used to subjectively probe whether participants adopted either the insight or the analytical strategy.

### Transcranial direct current stimulation

**Figure 3:**
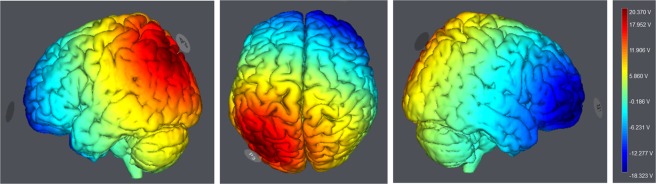
Simulation of the current density magnitude (V/m) generated by 2 mA a-tDCS to the lPPC (StimWeaver, Neuroelectrics, Spain). Left: sagittal view of the left hemisphere, Middle: superior view, Right: sagittal view of the right hemisphere.

All participants were equipped with tDCS (STARSTIM, Neuroelectrics) that included two saline-soaked sponge electrodes, and a small anodal electrode (25 cm^2^, current density 0.08 mA/cm2) to elicit a more focal stimulation and larger cathodal electrode (35 cm^2^) in order to avoid meaningful stimulation of the reference site. Theoretical simulation of the current density magnitude is illustrated by Fig. 3. The anode was localized over the lPPC (P3 based on 10–20 EEG system), while the cathode was placed over the right supraorbital region, referred to as Fp2. Hence, the anodal stimulation was concomitantly administered during the initial training or incubation period in the TrainStim and IncubStim groups, respectively. In both groups, the current was ramped up to reach 2 mA during the first 30 sec, and remained at this intensity for 19 min, and then reduced to 0 mA during the last 30 sec. For a high level of blinding, sham stimulation was delivered during initial training in the ShamStim group, and presented similar 30 sec up and down current modalities, but remained at 0 mA during 19 min.

### Data analysis

The main dependent variables were the response times to give the solution number (RTs) and the number of participants who gained insight solving strategy. Mean error rates of the solution number during the 3 blocks of the training session were also considered. The number of insight and analytical solvers was assessed using mean RTs of the solution number and debriefing reports. Participants were classified as insights solvers whenever they used the space bare systematically after the first or second response (R1 or R2), hence reflecting representational change of the NRT, which in turn drastically reduced the RTs to give the solution number. Analytical solvers corresponded to participant who did not use the space bare until the last response of the transformed string (R7), by continuously applying the two initial rules (i.e. same and different rules). This quantitative classification in the solver’s strategy using mean RTs was supported with qualitative data from individual debriefings. Specifically, insight solvers explicitly reported the use of a new and efficient rule to find the solution, while analytical solvers reported that they applied the two initial rules on each trial. For statistical analysis, chi-square 3 × 3 tables were used to determine whether a-tDCS delivered during either training (TrainStim vs. ShamStim groups) or incubation (IncubStim group) had a beneficial effect on insight solving strategy. Difference in time-point occurrence of insight was tested using a one-way ANOVA with the block occurrence of insight and group. Then, we performed a repeated measures ANOVA (ANOVA_RM_) with the mean RTs of the first training block and those obtained during the last practice block (i.e. either the verification block for insight solvers or the 10^th^ retest block for analytical solvers) as within-subject factor and solver strategy (insight vs analytical) as between-subject. Finally, an ANOVA_RM_ with group (TrainStim, ShamStim, and IncubStim) as between-subject factor and error rates (1^st^, 2^nd^ and 3^rd^ training blocks) as within-subject factor was performed. To do so, we excluded error rate data of insight solvers during the training session (n = 3). When appropriate, Bonferroni post-hoc tests were performed. We used Statistica workpackage 8 (StatSoft Inc, Tulsa, OK, USA) for statistical analysis. Throughout, the results are given as mean ± standard error of the mean, and a p < 0.05 was considered critical for assigning statistical significance.

## Results

**Figure 4:**
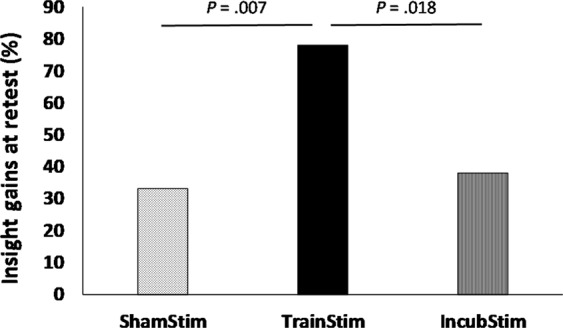
Insight solving strategy expressed in percent for the ShamStim, TrainStim and IncubStim groups. Data showed that a-tDCS over the lPPC applied early during problem solving drastically boosted representational change after a period of incubation, compared to when stimulation was delivered  during incubation.

The most striking result showed that stimulation over the lPPC during initial training on the NRT drastically boosted the occurrence of insight solving strategy compared to the other experimental conditions. In the TrainStim group, 17 participants out of 18 (94%) gained insight solving strategy, with 14 at retest (78%), compared to seven in the IncubStim group (39%, χ^2^ (1) = 5.60, *P* = 0.018; Fig. 4), and six in the ShamStim group (33%, χ^2^ (1) = 7.20, *P* = 0.007). Three participants of the TrainStim group elicited insight into the hidden rule during the third block of the training session, while all other participants found insight during the retest. The time-point of representational change in the NRT did not differ regarding the group of insight solvers (TrainStim, 4.8 ± 2.07; IncubStim, 6.3 ± 3.3; ShamStim, 4.1 ± 2.9; *P* = 0.33). Each participant who gained an efficient short cut in the RTs to give the correct solution (at R1 or R2), explicitly reported that they found the hidden rule, while the others reported applying the initial rules (i.e. same and different rules) on each NRT trial.

**Figure 5:**
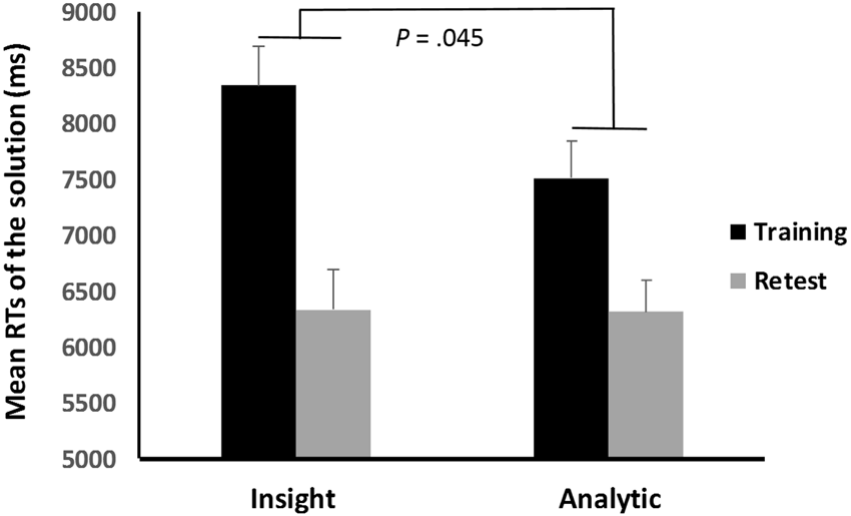
Mean RTs in insight and analytical solvers during the 1^st^ block of training and the last practice block. While there was no difference in the mean RTs to find the solution number during the 1^st^ block of training session, insight solvers showed a collapse in RTs when using the hidden rule strategy compared to analytical strategy. Noteworthy, the final block corresponds to the verification block of practice for insight solvers, while it was the last (i.e. 10^th^) retest block for analytical solvers.

There was no difference between insight and analytical solvers in the RTs to find the solution number during the 1^st^ training block (9.7 s ± 2.0 insight vs. 8.7 s ± 1.6 analytical, *P* = 0.05; Fig. 5), while RTs were significantly reduced in insight solvers who found the hidden rule (2.0 s ± 0.9 and 6.4 s ± 1.5 respectively, *P* < 0.001).

No group (F(2, 48) = 0.37, *P* = 0.68) or block (F(2, 96) = 1.51,  *P* = 0.22) difference was found when comparing error rates during the training session. Finally, it is unlikely that ineffective blinding of stimulation confounded the results, as we did not observe any difference in perceived sensations following excitatory and sham stimulations, hence supporting that participants were not aware of the experimental conditions (all *P* > 0.05).

Previous literature dealing with the NRT problem solving revealed that only 25% of participants on average gained insight when they practiced 13 continuous blocks^8,15^. On the other hand, the occurrence of insight solving strategy doubled when there was an incubation period of short wake quiescence^5^. Our data extend these findings in demonstrating that applying a-tDCS over the lPCC before incubation, but not during, strongly potentiated change in the representation of problem solving.

Overall, our findings did not reveal any peculiar behaviour related to the NRT characteristics before the occurrence of the representational change, but in general the participants reached a final solution earlier during the retest, regardless of the stimulation condition.

## Discussion

The purpose of the present study was to investigate whether a-tDCS over the lPPC during either training or incubation might boost change in the representation of problem solving. We found that applying a-tDCS over the lPCC early on the NRT, but not during incubation, strongly potentiated insight solving strategy. These data support that the lPPC is involved in the initial representation of a problem solving situation, and that stimulation of this region substantially potentiates subsequent change in the representation of the problem solving. It is well-established that the PPC is associated with attentional processes involving top-down attentional control and stimulus-driven attentional reorientation^25^. It is relevant to say that in the context of problem solving, the PPC contributes to the early capture of salient information, and the switch of attention toward spatially unexpected stimuli locations^26^. The depolarization induced by a-tDCS over the lPPC during the initial representation of the NRT might thus have enhanced the attentional efficiency to catch salient stimuli related to the structure of the problem solving (e.g. analogy between the second and the final responses), and to shift processes toward alternative solution strategy (e.g. from analytic to insight). This finding is in line with recent integrated framework on the PPC in adaptive visual processing, which includes selection, representation, and maintenance of salient visual information to guide thoughts^12^.

Our results further showed that delivering a-tDCS during incubation did not increase the occurrence of change in the representation of problem solving relative to the ShamStim condition. While this finding seems to slightly challenge previous data supporting effective *offline* a-tDCS delivery before verbal insight paradigm^19,27^, no study was designed to determine the most effective period for delivering a-tDCS in problem solving. Interestingly, Luft *et al*.^28^ recently reported that cathodal inhibitory tDCS over the left dorsolateral prefrontal cortex during 15 min of incubation was likely to promote solving of mismatch problems, though stereotypical insight solutions caused a floor effect. Based on the decrease of connectivity between left and right fronto-parietal regions elicited during default networks^29^, incubation in problem solving might benefit from cathodal inhibitory rather than anodal excitatory stimulation. Alternatively, accumulated evidence underlined the early contribution of the PPC during the *online* building of memory representation^30,31^, which may not remain relevant during *offline* incubation in problem solving. Instead, the prefrontal cortex has been extensively reported in spontaneous mental restructuring or updating processes, likely to prompt an abrupt emergence of explicit knowledge producing an insight^32,33^. Therefore, one can speculate that the prefrontal cortex might underpin effective incubation on problem solving, leading to a breakthrough of solution^34,35^. Exploring the nature of insight in problem solving led us to consider the functional interaction of regions mediating the cognitive control system (i.e. *online process during the task*) and the default network (*offline incubation*)^36,37^, both exhibiting an antagonistic relation during cognitive tasks and rest^38^. Overall, our data support that representational change during problem solving can be enhanced by means of brain stimulation, while the timing of delivery and the type of stimulation, as well as related brain targets, should be further considered in future investigations^1^.

As with all research, this study has some limitations that should be considered before drawing general conclusions. Although the a-tDCS montage did strongly stimulate the lPPC, few residual electrical fields were observed in visual areas. Thus, more research will need to clarify the specific role of the lPPC in insight problem solving, and should be performed using multifocal tDCS montage to avoid current transfer beyond the targeted area^39^. Caution should be also exercised when conceptually considering the accuracy of the insight moment using the NRT paradigm. The collapse in RTs, considered as the “Aha” phenomenon, might not reflect the exact moment of the representational change in the way to solve the task. We acknowledge that insight solvers might have found the hidden rule requiring to be “mentally” validated before its effective execution. Hence, the drop in RTs may rather correspond to the post-representational change occurrence. Our debriefing data mainly probed the type of strategy used on NRT, but did not enable to determine precisely whether or not the “aha” moment occurred before or during the short-cut in RTs. We suggest that further qualitative information during debriefings or questionnaire should resolve this issue. Finally, we did not assess psychometric abilities that might have influenced insight solving strategy such as fluid intelligence ability^40,41^. Future experiments should therefore consider this potential predictor of insight.

To conclude, early stimulation of the lPPC might contribute to substantially improve the encoding of salient information facilitating the processing of knowledge reorganization during incubation, hence promoting the occurrence of insight solving strategy. This study provides further evidence that the initial representation of problem solving supported by memory and attentional systems is a crucial step before leaving it aside to be incubated.
